# Plasma microRNA biomarkers for detection of mild cognitive impairment: Biomarker Validation Study

**DOI:** 10.18632/aging.100624

**Published:** 2013-12-22

**Authors:** Kira S. Sheinerman, Vladimir G. Tsivinsky, Laila Abdullah, Fiona Crawford, Samuil R. Umansky

**Affiliations:** ^1^ DiamiR, LLC, Princeton, NJ 08852, USA; ^2^ Roskamp Institute, Sarasota, FL 34243, USA

**Keywords:** microRNA, plasma, circulating, Mild Cognitive Impairment (MCI), Alzheimer's disease, neurodegeneration, brain, synapse

## Abstract

A minimally invasive test for early detection and monitoring of Alzheimer's and other neurodegenerative diseases is a highly unmet need for drug development and planning of patient care. Mild Cognitive Impairment (MCI) is a syndrome characteristic of early stages of many neurodegenerative diseases. Recently, we have identified two sets of circulating brain-enriched miRNAs, the miR-132 family (miR-128, miR-132, miR-874) normalized per miR-491-5p and the miR-134 family (miR-134, miR-323-3p, miR-382) normalized per miR-370, capable of differentiating MCI from age-matched control (AMC) with high accuracy. Here we report a biomarker validation study of the identified miRNA pairs using larger independent sets of age- and gender- matched plasma samples. The biomarker pairs detected MCI with sensitivity, specificity and overall accuracy similar to those obtained in the first study. The miR-132 family biomarkers differentiated MCI from AMC with 84%-94% sensitivity and 96%-98% specificity, and the miR-134 family biomarkers demonstrated 74%-88% sensitivity and 80-92% specificity. When miRNAs of the same family were combined, miR-132 and miR-134 family biomarkers demonstrated 96% and 87% overall accuracy, respectively. No statistically significant differences in the biomarker concentrations in samples obtained from male and female subjects were observed for either MCI or AMC. The present study also demonstrated that the highest sensitivity and specificity are achieved with pairs of miRNAs whose concentrations in plasma are highly correlated.

## INTRODUCTION

The importance of early diagnosis, treatment and prevention of Alzheimer's disease attracts the attention of scientific and medical communities, regulatory agencies, such as the US Food and Drug Administration (FDA), and industry and government leaders in many countries [1-3]. The number of AD patients and those in high risk populations grows quickly, especially in developed countries, due to increased lifespan. A number of investigational anti-AD drugs, targeting various processes characteristic of AD pathogenesis, have failed in recent clinical trials [1,4-6], likely due to massive neuronal loss and advanced stages of the disease in the enrolled patients [3-5]. It has been demonstrated that AD dementia is preceded by 10-20 years of the disease development, initially without clinical symptoms (pre-symptomatic AD), and then manifested as MCI [7-9]. It is important to note that the detailed analysis of failed clinical trials has demonstrated a therapeutic benefit in the sub-groups of patients with mild and moderate symptoms of AD [6, see also https://investor.lilly.com/releaseDetail.cfm?ReleaseID=702211 and http://www.alzforum.org/new/detail.asp?id=3288]. The high need for development of new methods for early AD detection is also emphasized in recent publications from the FDA [10, see also http://www.fda.gov/downloads/Drugs/GuidanceComplianceRegulatoryInformation/Guidances/UCM338287.pdf] and the U.S. Department of Health and Human Services (“National Alzheimer's Project Act”, available at: http://aspe.hhs.gov/daltcp/napa/). Since cognitive testing cannot identify patients in pre-symptomatic stages of AD, effective biomarkers are necessary for successful patient stratification and treatment monitoring [3-5].

Due to the Alzheimer's Disease Neuroimaging Initiative (ADNI) in the US (http://www.adni-info.org/) and similar projects in other countries, a significant progress in early detection of AD with high sensitivity and specificity by imaging techniques and analysis of protein biomarkers in cerebrospinal fluid has been achieved [7]. However, the high cost and invasiveness of these methods make their application for primary screening of large populations impractical [11]. Various approaches to the development of non-invasive or minimally invasive assays for early detection of AD have been tested [12-19]. Currently there is no reliable molecular test for diagnosing AD at the pre-symptomatic or MCI stage. Recently we proposed an approach for early detection of MCI based on analysis of cell-free circulating miRNAs in plasma by RT-qPCR [20]. Several innovations were demonstrated to be effective for selection of potential miRNA biomarkers. First, we hypothesized that changes in concentrations of circulating miRNAs enriched in the brain, and more specifically in hippocampus and frontal cortex, were more likely to reflect AD-associated pathologic processes in the brain than ubiquitous or other organ-enriched miRNAs. Second, we analyzed miRNAs present in neurites and synapses, dysfunction and destruction of which is characteristic of early stages of neurodegeneration, and therefore, could affect expression and secretion of these miRNAs. Third, to compensate for processes unrelated directly to MCI, e.g. changes in blood-brain barrier permeability, we used the “biomarker pair” approach [20-23] normalizing neurite/synapse miRNAs by other brain-enriched miRNAs, which could be expressed in brain areas or cell types not involved in early stages of AD and MCI, as well as miRNAs with levels in plasma changing differently when compared with neurite/synapse miRNAs. Two sets of biomarker pairs, miR-132 (miR-128/miR-491-5p, miR-132/miR-491-5p and miR-874/miR-491-5p) and miR-134 (miR-134/miR-370, miR-323-3p/miR-370 and miR-382/miR-370), capable of differentiating MCI from AMC with sensitivity and specificity of 79%-100% were identified. In a separate small longitudinal study, the identified biomarker miRNA pairs successfully detected MCI in a majority of patients at the asymptomatic stage 1-5 years prior to clinical diagnosis. These miRNA pairs also differentiated AD from AMC (P<0.001) and appeared effective in detecting age-related brain changes in younger and older controls. Thus, while biomarkers of miR-132 and miR-134 sets do not seem to be specific to AD, they detect some common processes (possibly neurite/synapse dysfunction and destruction), characteristic of AD and other neurodegenerative diseases, and are capable of detecting MCI early. The initial report described analysis of 30 plasma samples in each group (AMC, MCI and AD; 10 in the pilot study for miRNA selection, and 20 in the feasibility study); all plasma samples were collected at the Roskamp Institute (Sarasota, FL).

In the present biomarker validation study we analyzed new larger sets of gender- and age-matched plasma samples (50 MCI and 50 AMC) collected at different sites.

## RESULTS

### Biomarker validation

**Figure 1:**
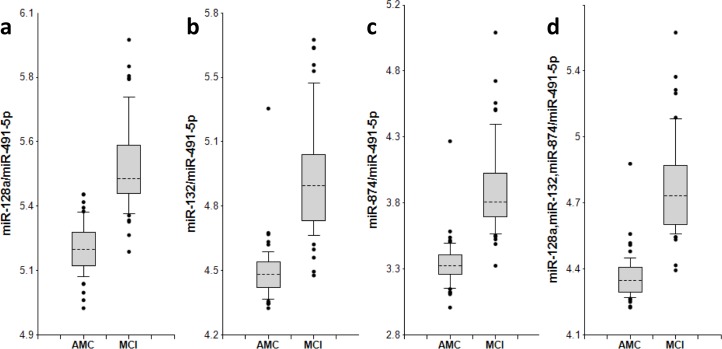
miR-132 family biomarker pairs in plasma of AMC and MCI subjects The concentrations of miRNA in plasma samples of MCI and age-matched donors with normal cognitive function, 50 samples in each group, were measured by RT-PCR and the ratios of various miRNA were calculated as 2^−ΔCt^ × 100. Here and in other figures with box and whisker plots the results are presented in the Log10 scale. The upper and lower limits of the boxes and the lines inside the boxes indicate the 75th and 25th percentiles and the median, respectively. The upper and lower horizontal bars denote the 90th and 10th percentiles, respectively. The points indicate assay values located outside of 80% data. AMC: age-matches controls; MCI: MCI patients.

**Figure 2:**
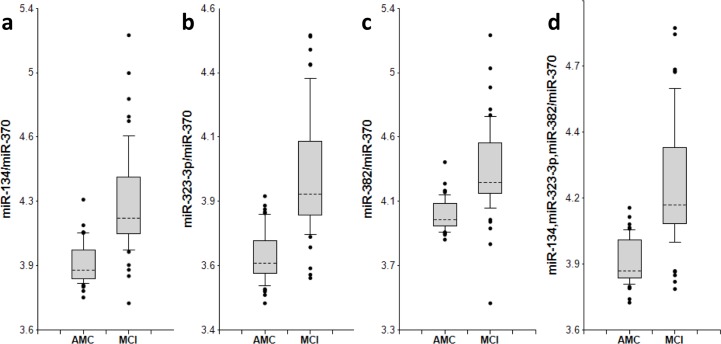
miR-134 family biomarker pairs in plasma of AMC and MCI subjects The concentrations of miRNA in plasma samples of MCI and age-matched donors with normal cognitive function, 50 samples in each group, were measured by RT-PCR and the ratios of various miRNA were calculated as 2^−ΔCt^ × 100. See the legend to Fig. 1 for the description of the box and whisker plots. AMC: age-matches controls; MCI: MCI patients.

**Figure 3:**
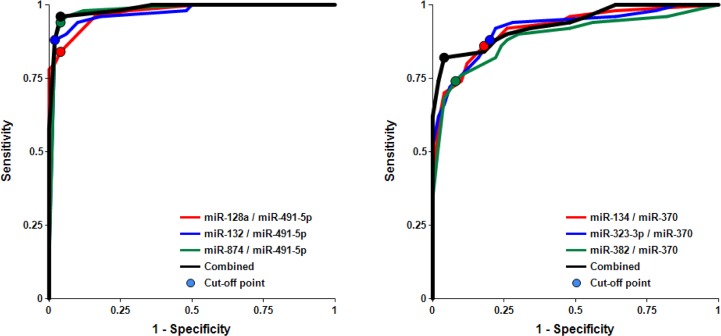
Receiver-Operating Characteristic (ROC) curve analysis of differentiation between MCI patients and age-matched controls obtained with different biomarker pairs The areas under the ROC curve (AUC), sensitivity, specificity and accuracy for each biomarker/normalizer pair presented in Table 2 are calculated for the “cutoff” point (indicated as a dot on each plot) – the value of the ratio of paired miRNA where the accuracy of predictions is the highest (see Materials and Methods).

The concentrations of 8 miRNAs were measured by RT-qPCR analysis in plasma samples from 50 MCI patients and 50 AMC subjects (Table 1). The ratios for miRNAs from the miR-132 family to miR-491-5p and for miRNAs from the miR-134 family to miR-370 (2^−ΔCt^) are presented as box-plots in Fig. 1 and 2, respectively. Fig. 3 presents Receiver-Operating Characteristic (ROC) curves for miR-132 and miR-134 families. The area under the ROC curve (AUC) for miR-128/miR-491-5p, miR-132/miR-491-5p and miR-874/miR-491-5p is 0.97, 0.97 and 0.98, respectively. These biomarker pairs (Set 1) differentiated MCI from AMC with 84%-94% sensitivity and 96%-98% specificity (Table 2). Further, biomarker pairs miR-134/miR-370, miR-323-3p/miR-370 and miR-382/miR-370 (Set 2) demonstrated 74%-88% sensitivity and 80-92% specificity (Table 2). AUC for miR-134/miR-370, miR-323-3p/miR-370 and miR-382/miR-370 are 0.92, 0.92 and 0.89, respectively. Combining biomarker miRNA pairs within the same set further improves sensitivity and specificity (Fig. 3 and Table 2). Combining biomarker miRNA pairs from miR-132 and miR-134 sets results in sensitivity and specificity that range between values obtained for the two sets of biomarker pairs.

**Table 1:** Demographics of plasma donors

Clinical Diagnosis	Number of Subjects	Age		Sex		MMSE
		Mean	Range	Male	Female	(mean±SD)
**AMC**	50	65.1	50-82	26	24	29.6 ± 0.6
**MCI**	50	68.2	51-82	21	29	26.0 ± 1.4

**Table 2:** Differentiation of MCI from AMC by miRNA biomarker pairs

Family/normalizer	miRNA	AUC	Sensitivity	Specificity	Accuracy	P-value (MCI vs. AMC)
miR-132/miR-491-5p	miR-128	0.97	84%	96%	90%	3.53E-16
	miR-132	0.97	88%	98%	93%	1.60E-15
	miR-874	0.98	94%	96%	95%	3.16E-16
	3 pairs	0.98	96%	96%	96%	1.51E-16
miR-134/miR-370	miR-134	0.92	86%	82%	84%	1.55E-12
	miR-323-3p	0.92	88%	80%	84%	9.46E-13
	miR-382	0.89	76%	90%	83%	5.37E-11
	3 pairs	0.93	80%	94%	87%	2.29E-12
miR-132/370	miR-128	0.80	62%	82%	72%	7.10E-7
	miR-132	0.82	74%	76%	75%	3.57E-8
	miR-874	0.85	88%	64%	76%	1.92E-9
	3 pairs	0.83	86%	66%	76%	1.30E-8
miR-134/miR-491-5p	miR-134	0.65	36%	88%	62%	1.00E-2
	miR-323-3p	0.63	38%	88%	63%	2.08E-2
	miR-382	0.63	38%	80%	59%	2.97E-2
	3 pairs	0.65	36%	88%	62%	1.82E-2

**Figure 4:**
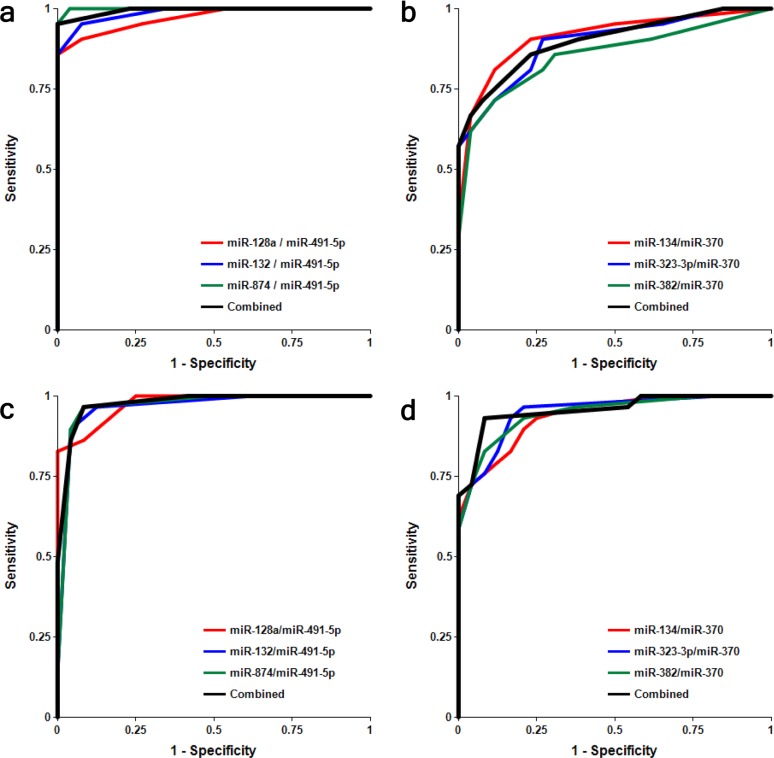
Receiver-Operating Characteristic (ROC) curve analysis of differentiation between MCI patients and age-matched controls obtained with different biomarker pairs in male (a, b) and female (c, d) cohorts The areas under the ROC curve (AUC), sensitivity, specificity and accuracy for each biomarker/normalizer pair presented in Table 3 are calculated for the “cutoff” point – the value of the ratio of paired miRNA where the accuracy of predictions is the highest.

It is important to analyze factors that could affect the test accuracy. The data presented in Figs. 4 and S1 and Table 3 show no statistically significant difference between female and male cohorts of AMC and MCI samples, although a trend toward slightly higher accuracy for MCI differentiation from AMC in the male cohort by miRNA pairs from the miR-132 family is observed, and the opposite trend is observed for miRNA pairs of the miR-134 family.

**Table 3:** Comparison of biomarker miRNA pairs of miR-132 and miR-134 families in male and female subjects

Male								
miRNA pair	128a/491-5p	132/491-5p	874/491-5p	miR-132 Fam. combined	134/370	323-3p/370	382/370	miR-134 Fam. combined
AUC	0.97	0.99	1.00	0.99	0.91	0.89	0.86	0.90
**Female**								
AUC	0.98	0.97	0.97	0.98	0.94	0.95	0.95	0.96
**Male – Female Comparison**								
P-value	0.763	0.479	0.201	0.686	0.601	0.315	0.167	0.285

### A role of miRNA normalizer

**Figure 5:**
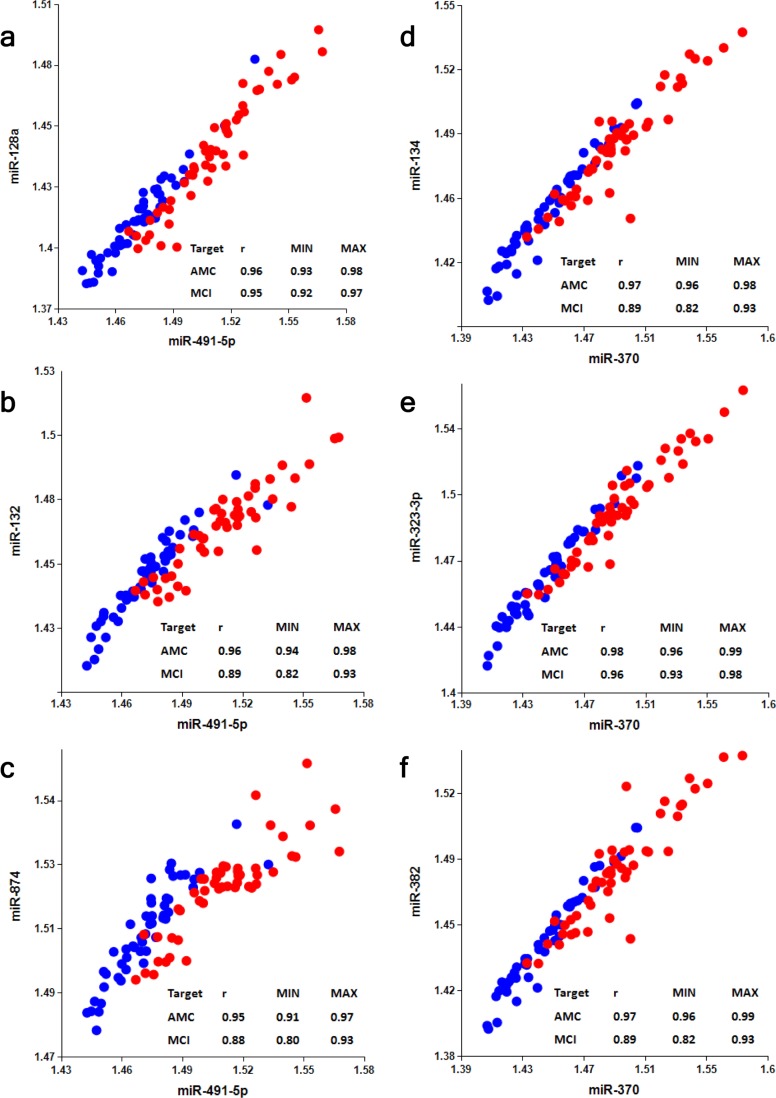
Analysis of correlation between members of miR-132 and miR-134 families and their optimal normalizers miR-491-5p and miR-370, respectively Spearman's rank correlation coefficient r along with 95% confidence intervals (MIN & MAX) is shown for AMC (blue dots) and MCI (red dots) subjects.

Selection of an optimal denominator (normalizer) for each miRNA family was shown to be essential [20]. miR-491-5p and miR-370 were found to be effective when paired with miRNAs of the miR-132 and miR-134 families, respectively. This finding has been further tested in the present study. Figs. S2-S4 and Table 2 show that if normalizers are switched between the two families, pairs miR-128/miR-370, miR-132/miR-370, miR-874/miR-370, miR-134/miR-491-5p, miR-323-3p/miR-491-5p and miR-382/miR-491-5p differentiate MCI from AMC with much lower sensitivity and specificity. Concentrations of miRNAs in plasma depend on numerous factors, including (i) levels of miRNA expression in various organs and tissues; (ii) levels of miRNA secretion from different cell types; (iii) stability of miRNAs in extracellular space and their appearance in plasma in different forms, such as exosomes and other micro-vesicles, complexes with proteins, lipids and, possibly, other molecules; and (iv) blood-brain barrier permeability for brain-enriched miRNAs. A pathological process may affect some or all of these factors. It is, therefore, logical to expect that a numerator and a denominator of an effective biomarker miRNA pair should share some of these basic common factors (e.g. both are brain-enriched and secreted in exosomes) and would change differently in response to a pathology). In such cases, one can expect a high correlation between miRNAs of miR-132 and miR-134 families and their optimal respective normalizers, miR-491-5p and miR-370. Data presented in Fig. 5 demonstrate that in the AMC cohort Spearman test r values for the correlation between miRNAs of the miR-132 family with miR-491-5p are in the 0.95-0.96 range and for the correlation between miRNAs of the miR-134 family with miR-370 are in the 0.97-0.98 range. In the MCI cohort, the correlation between the same miRNAs is slightly lower, indicating that the pathology differently affects plasma levels of miRNAs of the miR-132 family and of miR-491-5p, as well as levels of miRNAs of the miR-134 family and of miR-370. Correlations between neurite/synapse-enriched miRNAs from one family with the optimal normalizer of another family are significantly weaker (Fig. S5).

## DISCUSSION

The main objective of the present work was to validate previously identified sets of plasma biomarker miRNA pairs [20] in a larger study with clinical samples collected at sites different from the one used in the original study. The data have validated miRNAs of the miR-132 and miR-134 families, paired with miR-491-5p and miR-370 respectively, as highly sensitive biomarkers for detection of MCI. The overall accuracy for differentiating MCI from AMC is 90%-96% and 83%-87% for the biomarker miRNA pairs of miR-132 and miR-134 sets, respectively. The corresponding values obtained in the first feasibility study were 86% – 92% and 82% – 89%. Since a large number of MCI patients will progress to AD dementia [24-26], it is reasonable to suggest that these biomarker pairs detect early stages of AD as well, although they do not differentiate AD from MCI caused by other conditions. As was the case in the feasibility study, the miR-132 family biomarkers detected MCI with higher accuracy than the miR-134 family biomarkers. Although the roles of most miRNAs tested in this study in neuronal differentiation, function and pathology have not been elucidated yet, it has been demonstrated that miR-132 and miR-134 have opposite effect on neurons: miR-132 stimulates [27,28] and miR-134 [29] inhibits neurite growth. Also, the level of miR-132 has been shown to be lower in the hippocampus and temporal neocortex of AD patients [30,31]. Lau et al. [32] have demonstrated that downregulation of miR-132 occurs at Braak stages III and IV, prior to loss of neuron specific miRNAs. They have also found that deregulation of miR-132-3p in the AD brain appears to occur mainly in neurons displaying Tau hyper-phosphorylation and that the transcription factor FOX01a is a key target of miR-132 in the Tau network. Interestingly, the concentration of miR-128, which promotes neuronal maturation [33], has been shown to increase in the hippocampus in an intermediate stage and to decrease in a late stage of AD [34,35]. Aging-associated increase in the concentrations of miR-134 and miR-874 in serum has been demonstrated [36]. We plan to further analyze the utility of both sets of biomarker miRNA pairs for MCI detection in larger longitudinal studies.

The present study has not shown statistically significant differences between male and female cohorts in differentiating MCI from AMC, suggesting that a combined control group could be used in further studies. These results need to be confirmed in larger follow-on studies.

The present study further validated the use of effective “miRNA pairs”, i.e. pairing of an optimal miRNA normalizer (denominator in biomarker pair) with a particular miRNA as the numerator. In the previous study [20] we analyzed levels of neurite and/or synapse miRNAs and other brain-enriched miRNAs in plasma of MCI and AMC subjects, and then the ability of all possible miRNA pairs to differentiate MCI from AMC was tested. Neurite/synapse miRNAs (miR-132 and miR-134 families) were found to be the best nominators in the identified and selected biomarker pairs. These data supported our initial hypothesis: neurite/synapse miRNAs can be effective biomarkers of neuro-degeneration, because synapse dysfunction and subsequent neurite and synapse destruction are early events in the progression of neurodegenerative diseases. We also demonstrated that miR-491-5p was a preferred normalizer for the miR-132 family, and miR-370 was a preferred normalizer for the miR-134 family, although the nature of these preferences was not clear at the time. Here we have further analyzed this phenomenon and found that a high correlation between numerator and denominator of biomarker miRNA pair in plasma samples from different subjects is an important parameter for their compatibility. It is currently unclear on what factors such a correlation depends, since many factors likely affect concentrations of cell-free miRNAs in plasma. Intuitively, it seems reasonable to suggest that an efficient miRNA pair should include two plasma miRNAs, which share common properties (for example, miRNAs secreted/excreted by the same mechanism, miRNAs bound to the same protein in plasma or present in similar exosomes, etc.), but differ in their response to investigated pathology. Hence, correlation analysis could be a useful approach for selecting the effective biomarker pairs among bodily fluid miRNAs for various diagnostic applications.

Thus, the present study has validated two sets of plasma biomarker miRNA pairs for the early detection of MCI, providing a basis for a large longitudinal study for determining the biomarkers' ability to detect MCI and AD at pre-symptomatic stages. The described approach is complementary to other diagnostic technologies, such as neuroimaging and CSF analysis.

## MATERIALS AND METHODS

### Plasma samples

K2EDTA Plasma samples from 50 MCI patients and 50 AMC were obtained from a commercial vendor PrecisionMed (Solana Beach, California). The samples were collected in compliance with the Health Insurance Portability and Accountability Act (HIPAA) and a written consent was obtained from each subject. All samples were frozen at −20°C within 2 hours from collection, then transferred to −80°C, and stored and shipped at −80°C.

MCI diagnosis was based on several tests evaluating cognition: (i) ADAS-Cog (Alzheimer's Disease Assessment Scale-Cognitive subscale; (ii) CDRS (Clinical Dementia Rating Scale); (iii) Wechsler Memory Scale; and (iv) MMSE (Mini Mental State Examination). MCI classification requirements included the following parameters: (i) 28 ≥ MMSE ≤ 22 (ii) not demented; (iii) memory complaint; (iv) preserved general cognitive function; (v) intact activities of daily living (allowed problems with 2 or less of the following: phone calls, meal preparation, handling money, completing chores); (vi) abnormal memory function documented by scoring below the education adjusted cutoff on the Logical Memory II subscale (delayed paragraph recall) from the Wechsler Memory Scale–Revised (maximum score = 25): (a) < 8 for 16 years or more of education; (b) < 4 for 8-15 years of education; (c) < 2 for 0-7 years of education. Patients with other neurological disorders were excluded from the study.

Cognitive status of AMC subjects was also evaluated using metrics listed above. AMC subjects had MMSE scores of 29 or 30, maintained independent activities of daily living, and did not have a known history of neurological illnesses, psychiatric disorders, or other medical conditions that could potentially interfere with their cognitive performance.

Demographic characteristics of the study groups are summarized in Table 1.

### Plasma RNA extraction and qRT-PCR miRNA analysis

miRNA isolation and qRT-PCR analysis were performed by Asuragen Inc. (Austin, TX, USA) as previously described [20]. Briefly, RNA was extracted from 200 μl aliquots of plasma using Trizol treatment and silica binding. Single target qRT-PCR was performed using the TaqMan® Reverse Transcription Kit and miRNA specific stem-loop primers (Applied Biosystems, Foster City, CA, USA). The RT step was performed in triplicate and 2 μl plasma equivalents were present in final PCR.

### Bioinformatics analysis and statistical methods

All statistical calculations were performed with the use of custom software developed at DiamiR LLC (Princeton, NJ), as previously described [16]. Briefly, Mann-Whitney U-tests were used to evaluate significance of differentiation of any two patient groups by various miRNA pairs, and Spearman's rank correlation coefficient was calculated to estimate associations between various miRNAs. Receiver-Operating Characteristic (ROC) curves were constructed and the area under ROC curves (AUC) was calculated to evaluate sensitivity and specificity of various biomarker sets. The cutoff points on the ROC curves, at which accuracy of MCI detection is maximal, were selected.

## SUPPLEMENTAL MATERIALS


