# Supplementary material for: Plasma microRNA biomarkers for detection of mild cognitive impairment: Biomarker Validation Study
Source: Aging (Albany NY). 2013 Dec 22;5(12):925–38. doi: 10.18632/aging.100624 (PMC3883708; doi:10.18632/aging.100624)
Supplement: Supplementary file 1 [file aging-05-925-s001.pdf]

SUPPLEMENTAL MATERIALS

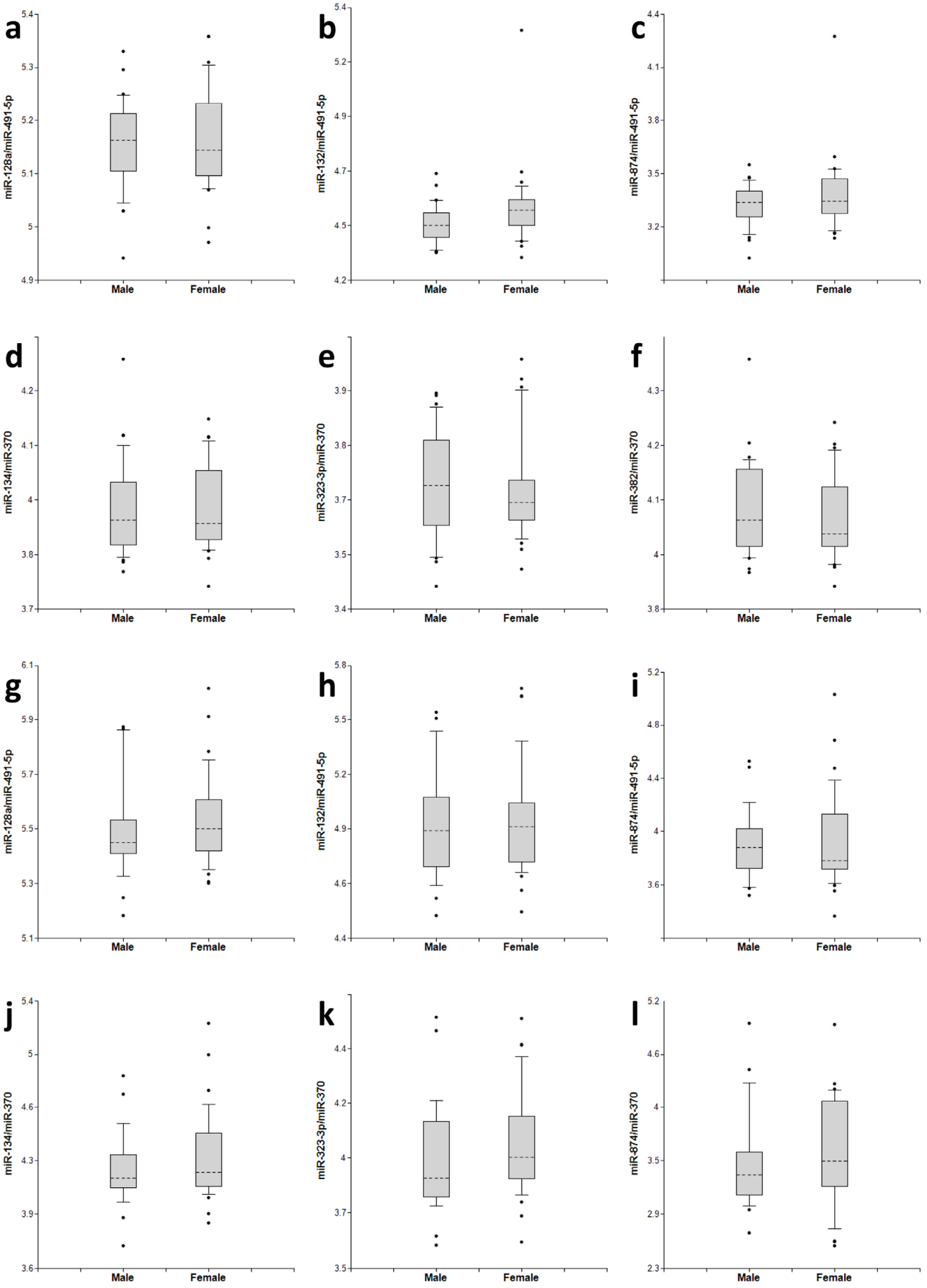

Figure S1. Comparison of two biomarker families in male and female subjects. **a-f**: AMC; **g-l** : MCI. The concentrations of miRNA in plasma samples of MCI and age-matched donors with normal cognitive function were measured by RT-PCR and the ratios of various miRNA were calculated as $2^{-\Delta Ct}$ x 100. Here and in other figures with box and whisker plots the results are presented in the Log10 scale. The upper and lower limits of the boxes and the lines inside the boxes indicate the 75th and 25th percentiles and the median, respectively. The upper and lower horizontal bars denote the 90th and 10th percentiles, respectively. The points indicate assay values located outside of 80% data.

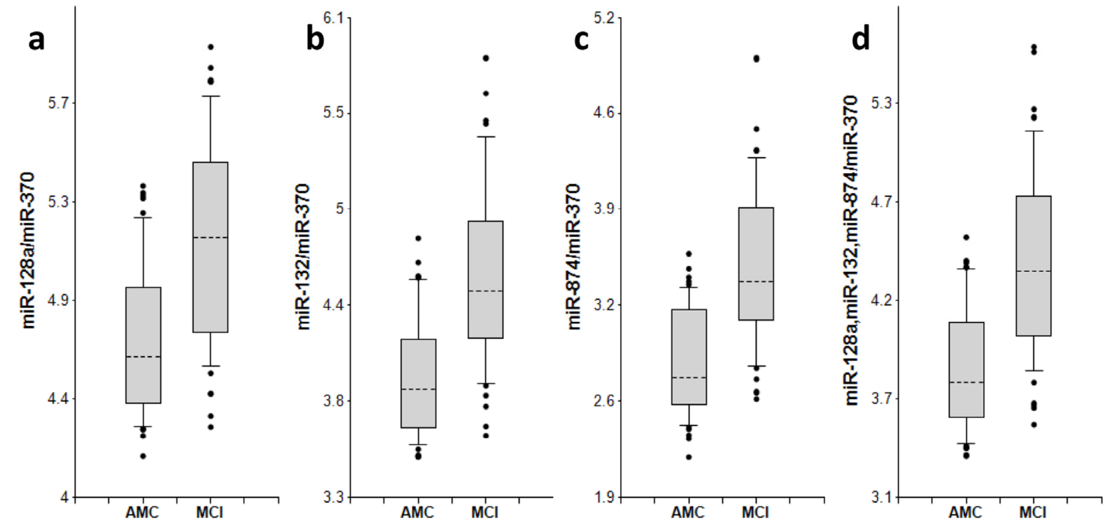

Figure S2. miRNAs of miR-132 family paired with miR-370 in plasma of AMC and MCI subjects. The concentrations of miRNA in plasma samples of MCI and age-matched donors with normal cognitive function, 50 samples in each group, were measured by RT-PCR and the ratios of various miRNA were calculated as $2^{-\Delta Ct}$ x 100. See the legend to Fig. S1 for the description of the box and whisker plots.

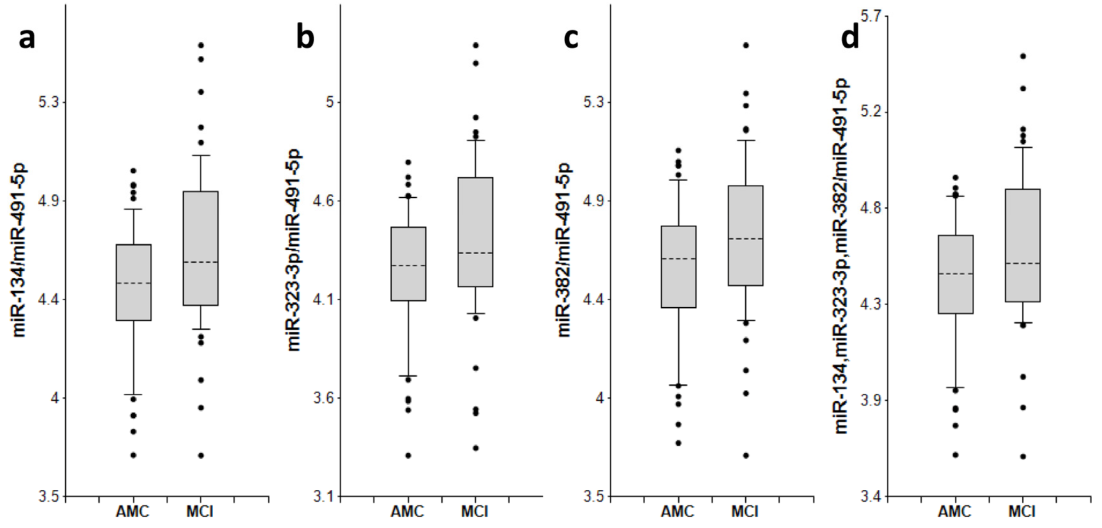

Figure S3. miRNAs of miR-134 family paired with miR-491-3p in plasma of AMC and MCI subjects. The concentrations of miRNA in plasma samples of MCI and age-matched donors with normal cognitive function, 50 samples in each group, were measured by RT-PCR and the ratios of various miRNA were calculated as $2^{-\Delta Ct}$ x 100. See the legend to Fig. S1 for the description of the box and whisker plots.

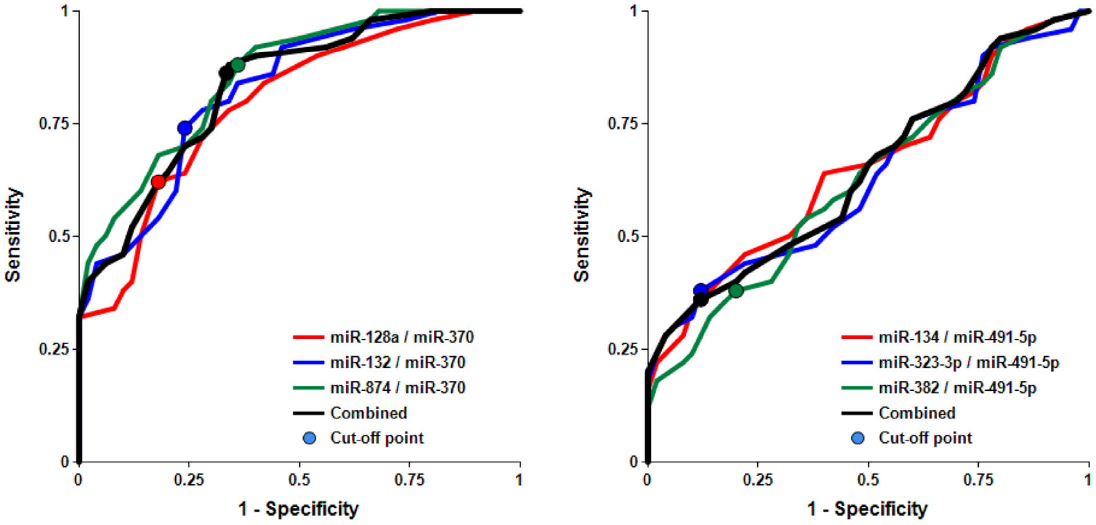

Figure S4. Receiver-Operating Characteristic (ROC) curve analysis of differentiation between MCI patients and age-matched controls obtained with miR-132 paired with miR-370 and miR-134 family members paired with miR-491-5p. The areas under the ROC curve (AUC), sensitivity, specificity and accuracy for each biomarker/normalizer pair presented in Table 2 are calculated for the “cutoff” point (indicated as a dot on each plot) - the value of the ratio of paired miRNA where the accuracy of predictions is the highest (see Materials and Methods).

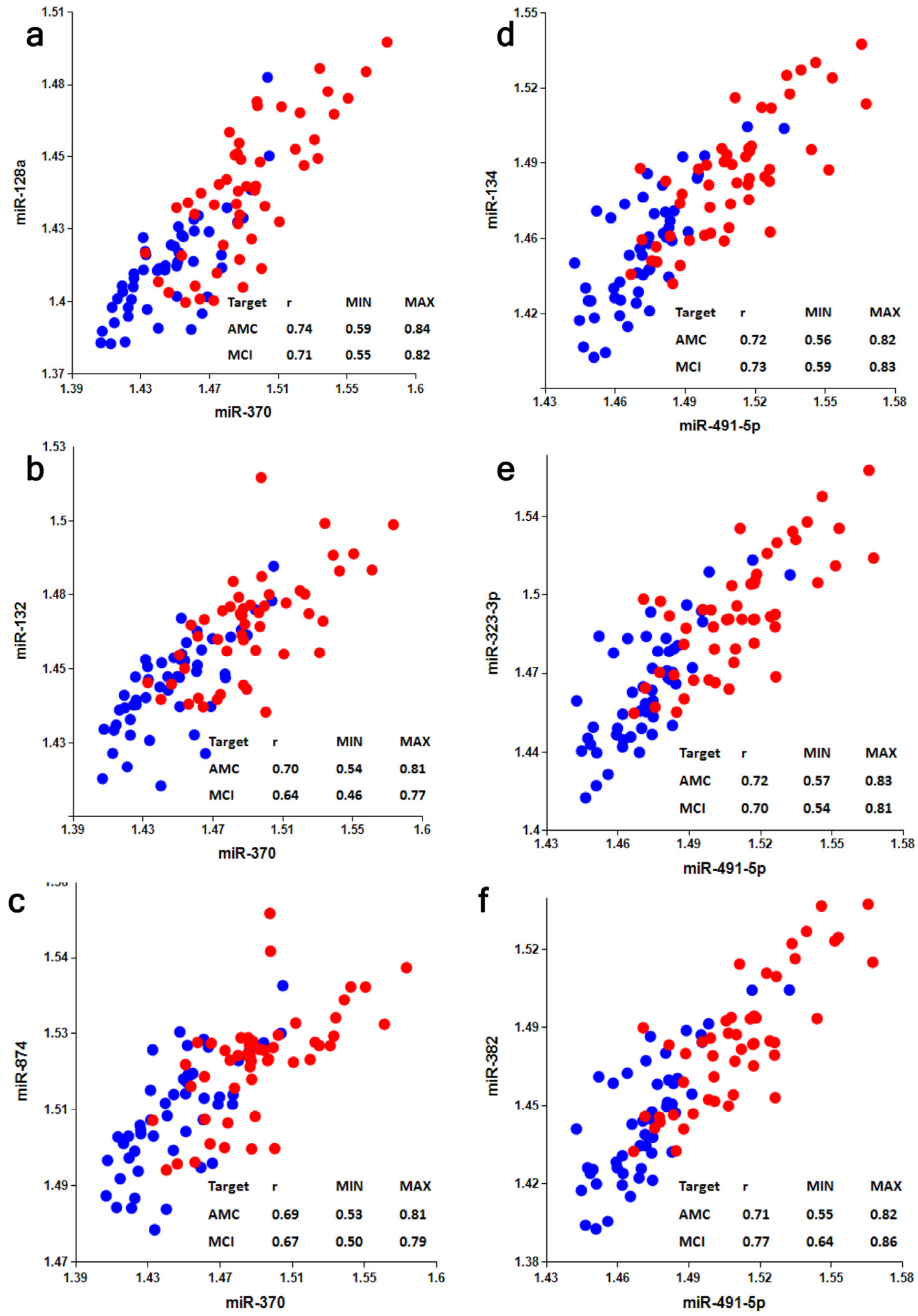

Figure S5. Analysis of correlation between members of miR-132 and miR-134 families and normalizers optimal for another family, miR-370 and miR-491-5p, respectively. Spearman's rank correlation coefficient r along with 95% confidence intervals (MIN & MAX) is shown for AMC (blue dots) and MCI (red dots) subjects.
